# Hepatitis B testing and treatment in HIV patients in The Gambia—Compliance with international guidelines and clinical outcomes

**DOI:** 10.1371/journal.pone.0179025

**Published:** 2017-06-14

**Authors:** Gibril Ndow, Mindy L. Gore, Yusuke Shimakawa, Penda Suso, Abdoulie Jatta, Saydiba Tamba, Amina Sow, Coumba Touré-Kane, Fouzia Sadiq, Saihou Sabally, Ramou Njie, Mark R. Thursz, Maud Lemoine

**Affiliations:** 1 Division of Digestive Diseases, Department of Surgery & Cancer, St. Mary’s Hospital Campus, Imperial College London, United Kingdom; 2 Hepatitis Unit, Disease Control & Elimination, MRC Unit The Gambia, Fajara, The Gambia; 3 Section of Virology, Department of Medicine, Imperial College London, London, United Kingdom; 4 Unité d’Épidémiologie des Maladies Émergentes, Institut Pasteur, Paris, France; 5 Laboratoire Bactériologie-Virologie, CHU Aristide Le Dantec, Université Cheikh Anta DIOP, Dakar, Senegal; 6 Hands on Care HIV Clinic, Brikama Health Centre, Brikama, The Gambia; 7 International Agency for Research on Cancer (IARC), WHO, Lyon, France; Centre de Recherche en Cancerologie de Lyon, FRANCE

## Abstract

**Background:**

Compliance with WHO guidelines on HBV screening and treatment in HIV-coinfected patients is often challenging in resource limited countries and has been poorly assessed in sub-Saharan Africa.

**Methods:**

Between 2015 and 2016, we assessed physician’s compliance with WHO guidelines on HIV-HBV coinfection in the largest HIV clinic in The Gambia, and the hepatic outcomes in HIV-HBV coinfected patients as compared to randomly selected HIV-monoinfected controls.

**Results:**

870 HIV-infected patients regularly seen in this clinic agreed to participate in our study. Only 187 (21.5%, 95% CI 18.8–24.3) had previously been screened for HBsAg, 23 (12.3%, 95% CI 8.0–17.9) were positive of whom none had liver assessment and only 6 (26.1%) had received Tenofovir. Our HBV testing intervention was accepted by all participants and found 94/870 (10.8%, 95% CI 8.8–13.1) positive, 78 of whom underwent full liver assessment along with 40 HBsAg-negative controls. At the time of liver assessment, 61/78 (78.2%) HIV-HBV coinfected patients received ART with 7 (11.5%) on Tenofovir and 54 (88.5%) on Lamivudine alone. HIV-HBV coinfected patients had higher APRI score compared to controls (0.58 vs 0.42, p = 0.002). HBV DNA was detectable in 52/53 (98.1%) coinfected patients with 14/53 (26.4%) having HBV DNA >20,000 IU/L. 10/12 (83.3%) had at least one detectable 3TC-associated HBV resistance, which tended to be associated with increase in liver fibrosis after adjusting for age and sex (p = 0.05).

**Conclusions:**

Compliance with HBV testing and treatment guidelines is poor in this Gambian HIV programme putting coinfected patients at risk of liver complications. However, the excellent uptake of HBV screening and linkage to care in our study suggests feasible improvements.

## Introduction

Chronic hepatitis B virus (CHB) infection is a major public health problem and a leading cause of morbidity and mortality globally. It affects approximately 250 million persons worldwide [1] and accounts for 650,000 deaths annually [2]. Without effective interventions and treatment, CHB infection will lead to an estimated 11.8 million deaths by 2030, primarily as a result of cirrhosis and hepatocellular carcinoma (HCC) [3]. Most of these deaths will occur in low-income countries (LICs) in sub-Saharan Africa (sSA) and Asia where CHB infection is endemic.

The World Health Assembly (WHA), in response to the increasing CHB–related morbidity and mortality, adopted a resolution to improve viral hepatitis prevention, testing and treatment worldwide [4]. Subsequently, the United Nations General Assembly and the World Health Organisation (WHO) respectively incorporated viral hepatitis elimination in the 2030 Agenda for Sustainable Development [5,6] and the WHO global health sector strategy on viral hepatitis 2016–2021 [3,7]. The WHO further recommends hepatitis B surface antigen (HBsAg) testing for all HIV-infected patients, and highly active antiretroviral therapy (HAART) containing at least two drugs effective against hepatitis B virus (HBV) for all HIV-HBV coinfected patients irrespective of disease stage or CD4 count [8], or where not feasible to treat patients with severe liver disease [9].

Whilst both HIV and HBV are endemic in sSA, the prevalence of HIV-HBV coinfection is highest in West Africa [10] where as many as 1 in 10 HIV-infected patients is coinfected with HBV. Uncontrolled HIV-HBV coinfection, due either to lack of or ineffective antiretroviral therapy (ART), increases the risk of death by more than twofold compared to HIV-monoinfected patients [11], irrespective of presence of liver disease [12]. Early and effective Tenofovir Disoproxil Fumarate (TDF) based ART, on the other hand, reduces risk of death among coinfected patients [10,11] underlying the need for implementing universal HBV testing and prompt ART treatment [8].

The Prevention of Liver Fibrosis and Cancer in Africa (PROLIFICA) programme in 2016 found high HIV and HBV prevalence among Gambian communities (2% and 8.5% respectively), with poor knowledge of hepatitis B and limited access to screening and treatment for HBV infection [13]. One of Africa’s smallest and poorest nations, The Gambia has one of the highest prevalence of HCC in West Africa [14] with two-thirds of these cases attributable to HBV infection [15]. In order to comply with the recent WHO goal for HBV elimination, we assessed the coverage of hepatitis B testing and treatment, and its impact on clinical outcomes in the largest HIV treatment centre in The Gambia, West Africa.

## Materials and methods

### Patients and study design

This cross-sectional study enrolled HIV infected patients aged ≥18years (range 22–63 years) registered at the Hands on Care HIV clinic in Brikama (The Gambia) who have had at least two successful clinic visits and baseline investigations. After written consent, we reviewed patient case notes to determine the coverage of hepatitis B surface antigen (HBsAg) testing for all HIV patients and use of TDF-based ART for known HIV-HBV coinfected patients. Thereafter, we screened the patients for HBsAg using the Determine^®^ rapid point-of-care kit (Alere^®^, USA). All HBsAg positive patients (HIV-HBV coinfected) and randomly selected HBsAg negative controls (HIV-monoinfected) had confirmatory HBsAg serology test (Architect HBsAg, Abbott, USA) and were further invited for complete liver assessment. Demographic and clinical information such as age, gender, past medical history, duration of HIV infection and antiretroviral drug exposure were collected, and a clinical examination and bedside ultrasonography performed. Liver transaminases (Vitros 350 Analyzer, Ortho Clinical Diagnostics, USA), haemoglobin and platelet count (CELL-DYN 3700 sample loader, Abbott, USA), HIV RNA (Abbott Real Time HIV-1 assay, 40copies/mL limit of detection), CD4+ T-cell count (flow cytometry) and in-house HBV DNA viral load quantification [16] were measured for all patients.

### Assessment of liver fibrosis

In accordance with WHO guidelines for setting without access a transient elastography device (Fibroscan), we assessed liver fibrosis using biochemical markers—aspartate aminotransferase (AST)-to-platelet ratio index (APRI) and FIB-4. APRI was calculated as [AST level (IU/L) / upper limit of normal for AST (IU/L) × 100/platelet count (10^9^/L)], and FIB-4 as [age (years) x AST (IU/L) / (platelet count (10^9^/L) x ALT (IU/L)^1/2^)]. Significant fibrosis defined was defined as APRI >1.5 [17] or FIB-4 >3.25 [18].

### HBV molecular analysis and polymerase gene sequencing

DNA was extracted from 500μL plasma using the QIAamp DNA Blood MiniKit (QIAgen, Germany). HBV DNA was quantified was by TaqMan based quantitative PCR assay with lower limit of detection of 50IU/L [16], using probe (200nM) sequence HBVTAQPR:5'FAM-CCTCTKCATCCTGCTGCTATGCCTCATC-3’MGBNFQ with forward and reverse primer (400nM) sequences HBVTAQ1:5'-GTGTCTGCGGCGTTTTATCA-3' and HBVTAQ2:5'-GACAAACGGGCAACATACCTT-3’ respectively [19]. Hepatitis B polymerase gene sequence was assessed in all participants with HBV DNA viral load ≥20,000IU/L.

The region from nucleotide 101 to 1149 of the HBV polymerase (pol) gene containing the highly conserved tyrosine-methionine-aspartate-aspartate (YMDD) motif was amplified by standard PCR using high fidelity polymerases (Supermix High Fidelity polymerase from Invitrogen or Q5^®^ High-Fidelity DNA Polymerase from New England BioLabs) and primers sense 5’-AYTGTCTCTTCCAYMTCRTC-3’ and antisense 5’-GGGGTAAAGGTTCAGRTAYTG-3’. Genotype of the amplified sequence was assessed using the NCBI genotyping tool [20].

### Statistical analyses

Demographic, clinical and virological characteristics of the study participants were presented according to their HBsAg status, and comparison made between HIV-monoinfected and HIV-HBV coinfected patients using the chi-squared test, Fisher’s exact test, or Wilcoxon rank-sum test. Factors associated with significant fibrosis (APRI >1.5) were identified using univariable logistic regression. As the number of participants with APRI >1.5 was small, multivariable analysis was not performed.

### Ethical approval

The MRC Scientific Coordinating committee and the joint Gambia Government/MRC Ethics committee approved this study (SCC1388). Consenting of participants followed group and individual sensitization, explanation of a study protocol and sufficient time for questions and discussion with spouse/family.

## Results

### Study population and compliance with guidelines

Between September 2015 and February 2016, 870 HIV-infected patients regularly seen at the Hands on Care HIV clinic were invited to participate in our study and all agreed to take part. The review of patient case notes revealed that only 187 (21.5%, 95% CI 18.8–24.3) had previously been tested for HBsAg, with 23 (12.3%, 95% CI 8.0–17.9) found to be HIV-HBV coinfected. None had a liver assessment based on liver transaminases, ultrasound, measurement of liver fibrosis or liver biopsy. Whilst all 23 patients previously known to be coinfected had been receiving ART, only 6 (26.1%, 95 CI 10.2–48.4) had received a TDF-based regimen.

**Fig 1:**
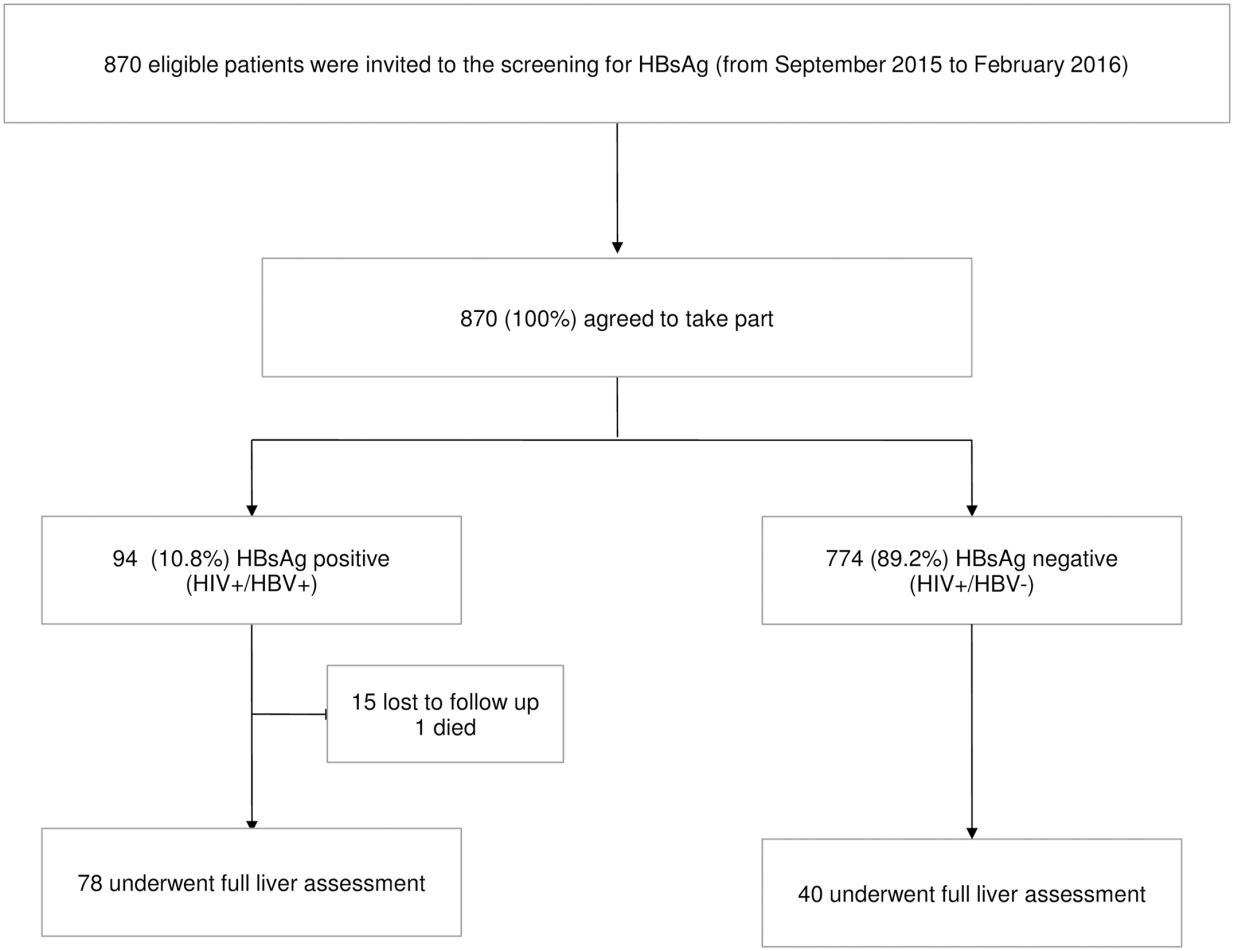
Flow diagram of the study population.

Of 870 patients accepted for the HBsAg screening, 94 tested positive giving a seroprevalence of 10.8% (95% CI 8.8–13.1). Of these, 15 (16%) were lost to follow up, and 1 died before the liver assessment. The remaining 78 HIV-HBV coinfected patients underwent full liver assessment, along with 40 randomly selected HBsAg negative controls (Fig 1).

Of the 78 coinfected patients who completed liver assessment as part of our study, 61 (78.2%, 95% CI 67.4–86.1) received ART, with 54/61 (88.5%, 95% CI 77.4–94.5) receiving Lamivudine (3TC) without TDF and only 7/61 (11.5%) receiving a TDF-based regimen. Median transaminase was within normal limits with only 5 patients (6.4%) having ALT above the upper limit of normal (ULN) and 1 (1.3%) with ALT >2xULN. Table 1 provides the characteristics of the study population by HBsAg status.

**Table 1:** Characteristics of the study population by HBV coinfection.

Parameters	HIV-HBV (n = 78)	HIV (n = 40)	P value
Median age (years, IQR)	38 (35–48)	42 (35–46)	0.5
Male sex (n, %)	8 (20)	16 (21)	0.9
Median BMI (Kg/m^2^, IQR)	21 (19–24)	22 (20–25)	0.4
HIV infection type (n, %) HIV1 HIV2 HIV1+2	58 (78) 3 (4) 13 (18)	34 (90) 2 (5) 2 (5)	0.2
Median duration since HIV diagnosis (months, IQR)	34 (14–68)	66 (20–82)	0.04
CD4+ T-cell count (n, %) <200 cells/mm^3^ ≥200 cells/mm^3^	24 (34) 46 (66)	13 (34) 25 (66)	1.0
Median CD4+ T-cell count (cells/mm^3^, IQR)	293.5 (147–444)	302 (170–411)	0.7
HIV RNA (n, %) Undetectable Detectable	30 (53) 27 (47)	26 (70) 11 (30)	0.09
Median HIV RNA (log copies/mL, IQR)	4.1 (3.2–5.0)	4.2 (3.2–4.6)	0.7
HBV DNA (n, %) Undetectable 50–2,000 IU/mL 2,000–20,000 IU/mL ≥2, 000 IU/mL	1 (1) 46 (60) 12 (16) 18 (23)	NA	NA
Median ALT (IU/L, IQR)	15 (12–25)	17 (11–26)	0.9
Median AST (IU/L, IQR)	28 (24–40)	24 (19–30)	0.004
Median platelet count (x10^9^/L, IQR)	186 (152–220)	198 (163–238)	0.1
APRI (n, %) <0.5 0.5–1.5 ≥1.5	29 (39) 38 (51) 7 (10)	27 (69) 12 (31) 0	0.005
Median APRI (IQR)	0.58 (0.43–0.88)	0.42 (0.35–0.53)	0.002
FIB-4 (n, %) <1.45 1.45–3.25 ≥3.25	30 (40) 39 (53) 5 (7)	25 (64) 14 (36) 0	0.03
Median FIB-4 (IQR)	1.72 (1.22–2.34)	1.35 (1.06–1.63)	0.004
ART regimen (n, %) Never treated 3TC without TDF TDF	17 (22) 54 (69) 7 (9)	5 (13) 29 (74) 5 (13)	0.5
Duration of 3TC exposure (n, %)*, ** <12 months 12–60 months ≥60 months	14 (23) 32 (52) 15 (25)	7 (21) 13 (38) 14 (41)	0.2

*This analysis only includes those who have ever received ART;

**3TC without a second HBV active anti-viral drug

ALT—alanine aminotransferase; APRI—AST to platelet ratio index; ART—antiretroviral therapy; AST—aspartate aminotransferase; BMI—body mass index; HIV—Human Immunodeficiency virus; RNA—ribonucleic acid; TDF—Tenofovir Disoproxil Fumarate; 3TC—Lamivudine

### Assessment of liver fibrosis

Compared to HIV-monoinfected patients, HIV-HBV coinfected patients had significantly higher median APRI (0.42 vs 0.58, p = 0.002) and FIB-4 (1.72 vs 1.35, p = 0.004), suggesting higher rate of liver fibrosis in coinfected patients. Table 2 shows the factors associated with significant liver fibrosis.

**Table 2:** Factors associated with significant liver fibrosis (APRI ≥1.5) in HIV-infected participants (n = 113).

	Prevalence of significant fibrosis (%)	Crude odds ratios	
		OR, 95% CI	P value
Age group <40 ≥40	5/58 (9) 2/55 (4)	1.0 0.4 (0.1–2.2)	0.3
Sex Women Men	6/90 (7) 1/23 (4)	1.0 0.6 (0.1–5.6)	0.7
BMI <25 ≥25	7/86 (8) 0/27 (0)	1.0 NA	0.1
Steatosis No Yes	6/105 (6) 1/8 (13)	1.0 2.4 (0.2–22.4)	0.5
HIV infection type HIV1 HIV2 HIV1+2	3/89 (3) 0/4 (0) 3/15 (20)	1.0 NA 7.2 (1.3–39.6)	0.02
HIV RNA Undetectable Detectable	0/53 (0) 6/37 (16)	NA	0.002
CD4+ T-cell count <200 cells/mm^3^ ≥200 cells/mm^3^	1/36 (3) 5/68 (7)	1.0 2.8 (0.3–24.7)	0.4
HBV co-infection No Yes	0/39 (0) 7/74 (10)	NA	0.04
ART Never 3TC without TDF TDF	2/19 (11) 5/82 (6) 0/11 (0)	1.0 0.6 (0.1–3.1) NA	0.5

### HBV molecular virology in 3TC exposed patients

Of 54 coinfected patients who were treated with 3TC without TDF, after a median duration of 28 months (IQR 12–60) on 3TC, HBV DNA and HIV RNA were detectable in 52/53 (98.1%, 95% CI 87.1–99.8) and 15/38 (39.5%, 95% CI 24.8–56.3) respectively. 14 of 53 (26.4%, 95% CI 16.0–40.3) had HBV DNA >20,000 IU/L. We successfully sequenced the region of the polymerase gene containing the YMDD motif in 12 of these 14 patients. 10 (83.3%) had M204V/I mutations present, with 8 (66.7%) having additional compensatory mutations at rtL180M and 3 (25.0%) at both rtL180M and rtV173L. Phylogenetic analysis of the polymerase gene sequence showed all but one patient (HBV genotype A) were infected with HBV genotype E.

We assessed the joint effect of HBV co-infection and 3TC resistance mutations on significant fibrosis (APRI >1.5) in HIV-infected patients. Compared to mono-infected, the prevalence of significant fibrosis was higher in those coinfected, and especially in those with 3TC resistance mutations. While no HIV-monoinfected patients (0/39) had significant fibrosis, 6/66 (9%) of patients coinfected with wild type HBV and 1/8 (13%) coinfected with resistant HBV strain had significant fibrosis (p for trend 0.05).

## Discussion

In The Gambia, a West African country highly endemic for HBV infection [13], our study provides two key messages: 1) poor compliance with the WHO hepatitis B testing and treatment guidelines [9] is poor with only 21.5% of HIV patients systematically screened for HBV infection and less than 15% on TDF-based ART, which puts HIV patients at risk of liver complications; 2) testing and linkage to care for HBV infection in HIV patients is accepted and feasible in HIV care programme in The Gambia.

To date, the compliance with the WHO guidelines [8,9] on the management of HBV and HIV coinfection in sSA—a region with high HIV-HBV coinfection prevalence [10], has been poorly documented. We found that in 2016, despite strong international recommendations and increasing global commitment to HBV elimination, HBsAg testing and effective HBV treatment remains poor in the largest Gambian HIV facility predisposing coinfected patients to severe clinical and virological outcomes, increased risk of liver-related morbidity, and diminished benefits of ART.

In our study, none of the 870 HIV-infected patients refused to be screened for HBV and almost 85% of HBsAg positive participants accepted to undergo liver assessment. This result is important since it suggests that HBV screening and linkage to care for HBV infection may be widely accepted in HIV-infected patients and feasible in HIV care facilities in sSA. This needs to be confirmed on a larger scale.

Whilst some countries in sSA successfully adopted a TDF-based first-line ART regimen in HIV reference centres [21], indication to treat and choice of ART regimen in Gambian HIV clinics was primarily determined by the type and stage of HIV infection. As a result, 3TC, a cheap and well-tolerated nucleoside analogue has been the backbone of first line ART in The Gambia since its introduction over a decade ago [22]. Although 3TC is an effective antiviral drug, it is associated with an increased risk of HBV resistance mutations following 12 to 24 months of monotherapy in patients with HBV infection [23]. Unsurprisingly, HBV DNA was detectable in almost all our study patients (98.1%) who received prolonged 3TC without TDF.

We found significantly higher prevalence (83.3%) of 3TC associated HBV resistance mutations compared to the 14.2% reported in a similar Gambian HIV-HBV cohort six year earlier who received 3TC without a second anti-HBV drug [24], as well as the 29.3% and 8.7% reported in a Ghanaian [25] and Ivorian [26] coinfected patients respectively. Our findings confirm that widespread prolonged usage of 3TC monotherapy in ART poses diagnostic and management problems to individual patients as well as a potential public health risk.

The high proportion of patients with detectable HIV viral load and low CD4 count after many months of ART, as well as the detectable HBV DNA among almost all coinfected patients receiving TDF in our study could be the result of unsatisfactory adherence to therapy, which might contribute to higher levels of HBV viremia and consequently to higher fibrosis and HBV resistance. Another explanation could be the deteriorating HIV services in The Gambia since 2007 when its former president claimed to have found herbal cure for HIV infection. This claim disrupted decades of HIV campaign and negatively impacted vital HIV services and research partnerships resulting in significant reduction in competent personnel and services in HIV facilities and suboptimal HIV care with frequent medication stock outs and several patients having their ARV regimens switched often. These underline the urgent need to improve HIV services and drug provision in Gambian HIV facilities.

HBV co-infection increases liver fibrosis [25, 27–31] as well as both all cause and liver related mortality [11,32], often in the absence of any evidence of liver disease [12]. Although our study lacked statistical power to show definitively a positive relationship between 3TC resistance mutation and increase in liver fibrosis, we show a trend towards such relationship (p = 0.05). Prompt initiation of TDF-based ART has been shown to reduce HBV viral replication and mortality in European [33] and Africa HIV-HBV cohort [11,12], further strengthening the need for compliance with the WHO HBV testing and treatment guidelines in Africa. It is worth noting that the uptake of TDF in first-line ART regimens in sSA is poorly documented. Our study shows that until 2016, only a minority of HIV patients in The Gambia received HBV testing and an even lesser proportion (7/78) of HIV-HBV coinfected patients received TDF. None of the patients had benefitted from a liver assessment even on the basis of liver transaminases. Indeed the local laboratory at this HIV facility, and almost all other HIV facilities in The Gambia, do not have the capacity to assess liver biochemistry. The 2016 consolidated HIV guidelines [8] recommending TDF-based ART for all HIV patients irrespective of degree of liver disease are thus well adapted to resource limited African settings, and African HIV facilities need to be supported to implement these guidelines.

Our study has a few limitations: first, we analysed a limited number of HIV-HBV coinfected patients; second, we assessed liver fibrosis using APRI score as recommended by the WHO for resource-limited settings without access to Fibroscan, which is the case for most HIV facilities in Africa where Fibroscan is rarely available outside research settings. The reliability and usefulness of platelet-based biomarkers like APRI for assessing liver fibrosis in HIV patients in Africa has been questioned [34] mainly because thrombocytopenia is commonly associated with advanced HIV infection. Third, our study did not assess for hepatitis C virus (HCV) and hepatitis D virus (HDV) coinfection. However, previous studies in The Gambia confirmed very low prevalence rates of both HCV and HDV infections [13]. Finally, our study suggests that adherence to ART was not satisfactory but we did not directly assess adherence to ART.

## Conclusion

Compliance with international HBV testing and treatment guidelines for HIV-infected patients is poor in this Gambian HIV programme, resulting in a low coverage of HBV testing, prolonged treatment with 3TC without TDF for coinfected patients, and poor HBV viral suppression with increased risk of HBV resistance and liver complication. However, interventions to improve HBV screening and linkage to care for HBV infection in HIV facilities are feasible.

## Supporting information

S1 File Data underlying the findings described in the manuscript. (XLSX)
